# Supplementary material for: GLP-1 Release by Rare Sugar D-Allulose Ameliorates Sucrose-Induced Obesity and Glucose Intolerance in Ovariectomized Mice
Source: Int J Mol Sci. 2026 Feb 8;27(4):1651. doi: 10.3390/ijms27041651 (PMC12940309; doi:10.3390/ijms27041651)
Supplement: Supplementary file 1 [file ijms-27-01651-s001.zip › ijms-4109223-supplementary.pdf]

Supplemental Figure and figure legends

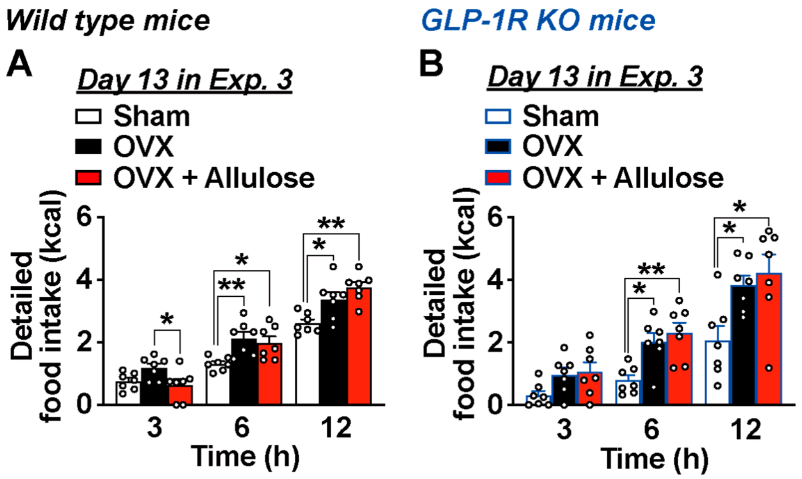

Figure S1.

Cumulative food intake up to 12 hours after oral gavage of D-allulose on Day 13 in Experiment 3.

Each genotype was divided into three groups: Sham (white bars), OVX control (black bars), and OVX D-allulose-treated (red bars). On Day 13 at 7:30, mice received water or D-allulose (3 g/kg) by oral gavage. n = 7. *p < 0.05, **p < 0.01 by one-way ANOVA followed by Tukey's test.

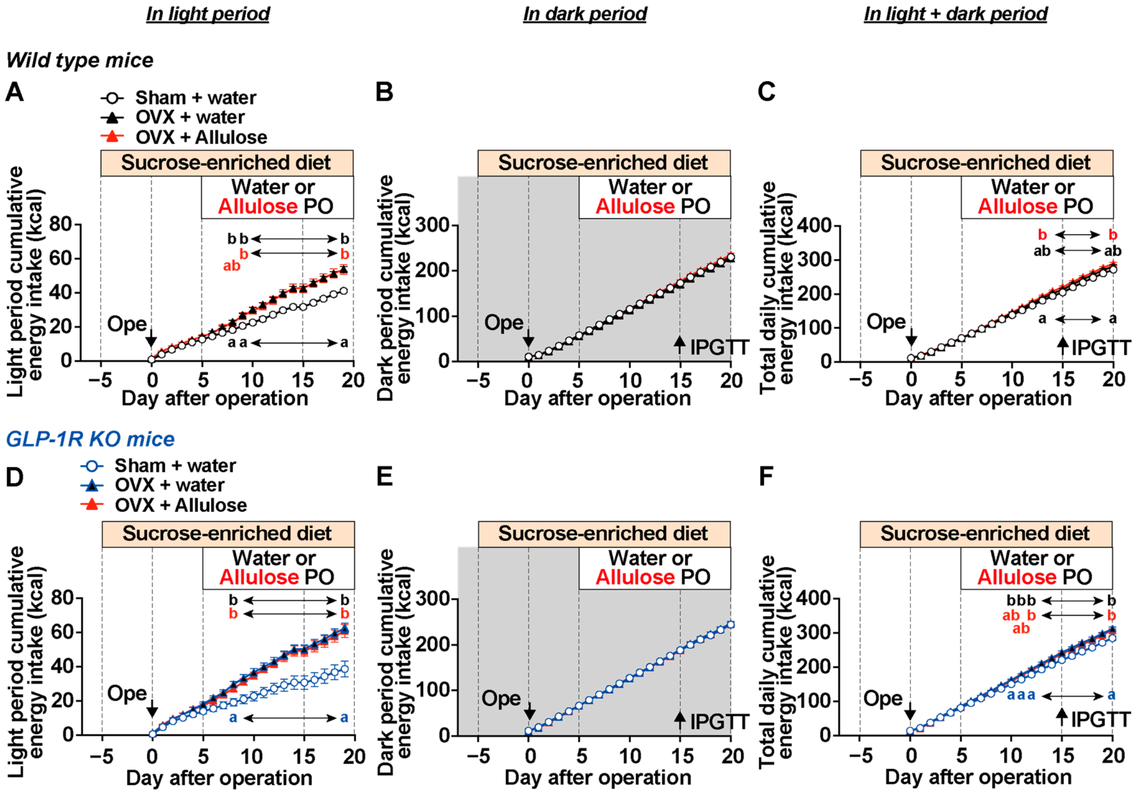

Figure S2.

Cumulative food intake during the light phase, dark phase, and over 24 hours during Experiment 3.

In Experiment 3, food intake during the light and dark phases was measured for 20 days after ovariectomy or sham surgery in wild type (**A–C**) and GLP-1 receptor knockout (GLP-1R KO, **D–F**) mice. From 5 days before surgery (Day −5), mice were switched from the CE-2 diet to a sucrose-enriched diet. From Day 5, mice received once-daily oral gavage of water or D-allulose at 3 g/kg for 2 weeks at onset of light phase. Time course of cumulative energy intake during light period (**A, D**), cumulative energy intake during dark period (**B, E**) and total daily cumulative energy intake over whole day (24 h, **C, F**). n = 7. Different letters indicate significant differences with $p < 0.05$ determined by two-way ANOVA followed by Tukey's test in **A–F**.

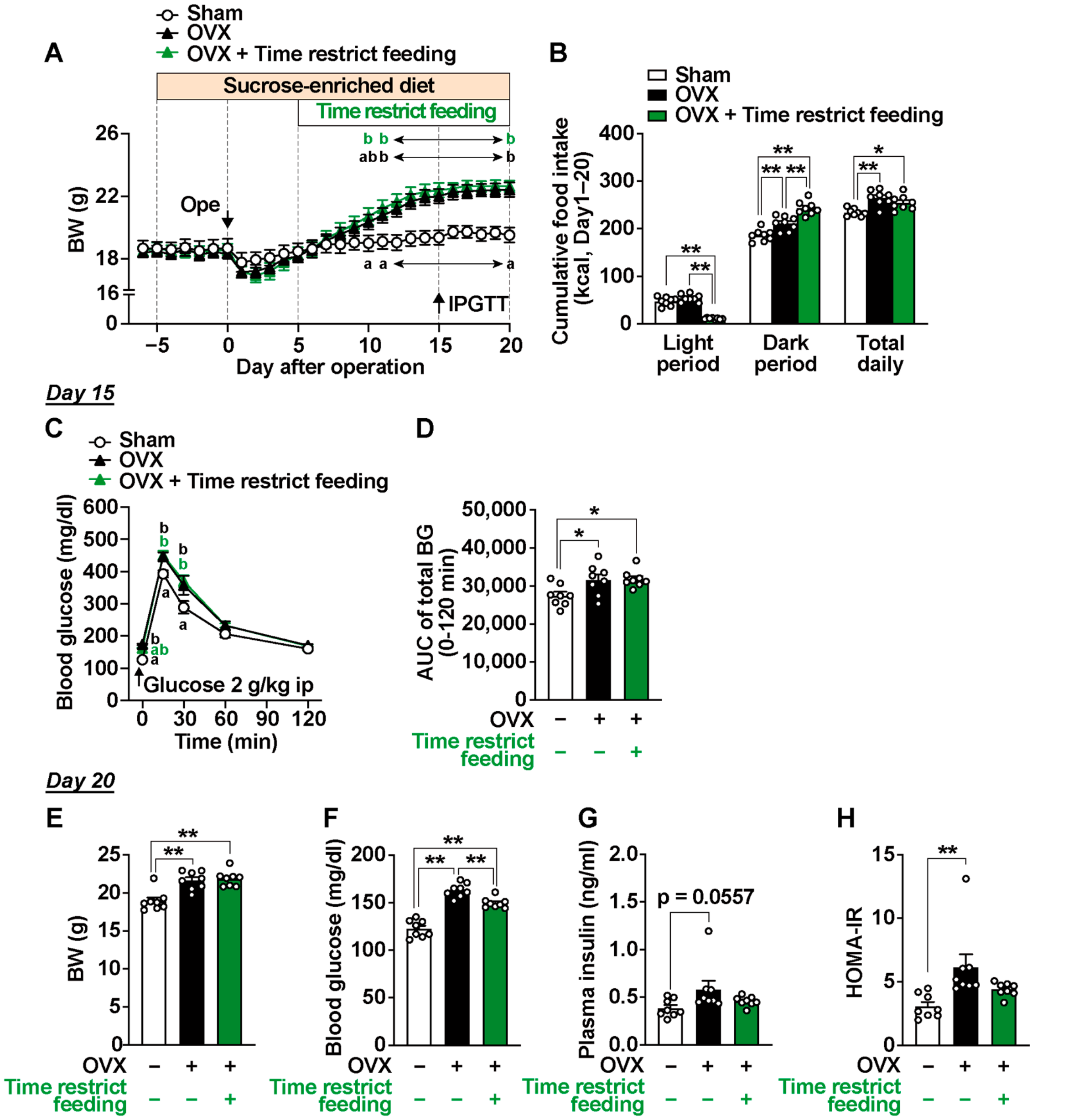

Figure S3.

Effects of light-phase fasting from Day 5 on ovariectomy- and sucrose-induced light-phase hyperphagia and body weight gain (Experiment 4).

Body weight and daily food intake were monitored for 20 days after ovariectomy or sham surgery in C57BL/6J mice. From 5 days before surgery (Day −5), mice were switched to a sucrose-enriched diet containing sucrose equivalent to 25% of the total diet weight, as in Experiment 3. From Day 5, the time-restricted feeding group was subjected to light-phase fasting. Time course of body weight (**A**); cumulative food intake during the light phase, dark phase, and total period from Day 1 to Day 20 (**B**); time course of blood glucose levels during the IPGTT and the AUC for blood glucose from 0 to 120 min on Day 15 (**C**, **D**); body weight, blood glucose, plasma insulin levels, and HOMA-IR on Day 20 (**E**–**H**). n = 8. Different letters indicate significant differences ($p < 0.05$) determined by two-way ANOVA followed by Tukey's post hoc test in **A** and **C**. *$p < 0.05$, **$p < 0.01$ by one-way ANOVA followed by Tukey's post hoc test in **B**, **D**, and **E**–**H**.
